# Poly(Dopamine)-Assisted Immobilization of Xu Duan on 3D Printed Poly(Lactic Acid) Scaffolds to Up-Regulate Osteogenic and Angiogenic Markers of Bone Marrow Stem Cells

**DOI:** 10.3390/ma8074299

**Published:** 2015-07-14

**Authors:** Chia-Hung Yeh, Yi-Wen Chen, Ming-You Shie, Hsin-Yuan Fang

**Affiliations:** 1 3D Printing Medical Research Center, China Medical University Hospital, Taichung City 40447, Taiwan; E-Mails: patrick6226@gmail.com (C.-H.Y.); evinchen@mail.cmuh.org.tw (Y.-W.C.); eviltacasi@gmail.com (M.-Y.S.); 2 Department of Thoracic Surgery, China Medical University Hospital, Taichung City 40447, Taiwan; 3 School of Medicine, College of Medicine, College of Public Health, Taichung City 40447, Taiwan

**Keywords:** poly (lactic acid), dopamine, 3D printed-scaffold, tissue engineering, osteogenic, angiogenic

## Abstract

Three-dimensional printing is a versatile technique to generate large quantities of a wide variety of shapes and sizes of polymer. The aim of this study is to develop functionalized 3D printed poly(lactic acid) (PLA) scaffolds and use a mussel-inspired surface coating and Xu Duan (XD) immobilization to regulate cell adhesion, proliferation and differentiation of human bone-marrow mesenchymal stem cells (hBMSCs). We prepared PLA scaffolds and coated with polydopamine (PDA). The chemical composition and surface properties of PLA/PDA/XD were characterized by XPS. PLA/PDA/XD controlled hBMSCs’ responses in several ways. Firstly, adhesion and proliferation of hBMSCs cultured on PLA/PDA/XD were significantly enhanced relative to those on PLA. In addition, the focal adhesion kinase (FAK) expression of cells was increased and promoted cell attachment depended on the XD content. In osteogenesis assay, the osteogenesis markers of hBMSCs cultured on PLA/PDA/XD were significantly higher than seen in those cultured on a pure PLA/PDA scaffolds. Moreover, hBMSCs cultured on PLA/PDA/XD showed up-regulation of the ang-1 and vWF proteins associated with angiogenic differentiation. Our results demonstrate that the bio-inspired coating synthetic PLA polymer can be used as a simple technique to render the surfaces of synthetic scaffolds active, thus enabling them to direct the specific responses of hBMSCs.

## 1. Introduction

Therapy for bone fractures due to trauma or resection presents unique challenges, due to the complex and three-dimensional (3D) geometry of the bone tissue [1,2]. The aim of tissue engineering was developing biological materials that restore, maintain, or enhance harmed tissue and organ regeneration, and it has been intensively studied in the past few decades [3,4,5]. Traditional methods of fabricating 3D porous scaffolds, such as polyurethane foam, porogen templating, solvent casting and freeze drying, were very difficult to control the porosity and interconnection of the scaffolds [6]. Recently, more ideal porous scaffolds with better control of pore morphology, pore size and porosity were developed and fabricated by the 3D printing technique. In brief, the CAD/CAM file was the basis for sets that can be a computer tomography or magnetic resonance morphology of the defect region, which are used to generate a 3D model that is then converted into a sequence of slices that are used to fabrication the corresponding real 3D object in layer-by-layer fashion [7]. In addition, 3D printing was used to fabricate various versatile free-form structures by a high flexibility in geometry and material [2,8]. Several studies have used different 3D-printing techniques to develop scaffolds using biocompatible materials such as collagen [8], poly-caprolactone [2], hydroxyapatite and tricalcium phosphate [9,10]. More recently, the pure PLA is a typical hydrophobic polymer material, which has a lack of cell-recognition signals and limited use in biomaterials [11,12].

The simple method for materials surface modification based on the mussel-inspired polydopamine (PDA) was proved by Messersmith’s group, and it has since been applied in a wide range of biomaterials applications [13,14]. Lots of studies influenced by the adhesion of mussels to rocks in wet environments have reported that the adhesive proteins secreted by mussels mainly contain dihydroxyphenylalanine (DOPA) and lysine, and this has attracted great attention in the field of biomedical materials [15]. Similarly, dopamine (DA) contains the same catechol functional group as that of the side chain of DOPA residues, as well as the same amine functional group, and a unique feature of PDA is its ability to deposit on various hydrophobic or hydrophilic surfaces via self-polymerization by the oxidation of DA in a weak alkaline buffer solution [16]. The material-independent PDA coating can obtained quickly by polymerization of DA, and the PDA layer serves as a platform for modification, containing spontaneous deposition of ceramic, metal, and other materials, as well as covalent immobilization of several serum adhesive proteins [17,18,19].

Several bone growth factors, including fibroblast growth factor, bone morphogenetic protein, platelet-derived growth factor, and transforming growth factor have been proved to be potential stimulators of bone regeneration [20,21]. One study has found an alternative bone growth factor from natural products to supersede these expensive growth factors, and traditional Chinese medicine has proved to be an ideal hunting ground [22]. Several compounds isolated from the leaf and stem of plants are prepared as powders for clinical use in Chinese herbal medicine and they have been shown to have beneficial clinical effects in recent years [23,24]. Dipsacus asperoides C.Y. Cheng et T.M. Ai is a perennial herb and the roots of D. asperoides, also named Xu Duan (XD) have been used in Traditional Chinese Medicine for hundreds of years as an antiosteoporosis, tonic and antiaging agent for the therapy of low back pain, traumatic hematoma, threatened abortion and bone fractures [25]. XD is commonly used as a major constituent of prescriptions for the treatment of bone diseases and functions in strengthening bone and healing bone fractures. Recent studies have confirmed that Dipsaci radix extract can increase bone density and alter bone histomorphology in mice [26] and has an osteoprotective effect in ovariectomized mice [25].

The objective of this study was to develop a simple procedure for PDA-assisted coating and XD immobilization on the 3D printed PLA scaffolds. The polymer was incorporated into dopamine coatings, resulting in a simple one-step coating procedure. The deposited PDA and XD coated films were examined by X-ray photoelectron spectroscopy (XPS), and their efficacy in accelerating adhesion of human bone-marrow mesenchymal stem cells (hBMSCs) was evaluated. Finally, the proliferation, osteogenesis and angiogenesis of hBMSCs were investigated to evaluate the efficacy of the surface modification.

## 2. Materials and Methods

### 2.1. Fabrication of PLA Scaffolds

**Figure 1:**
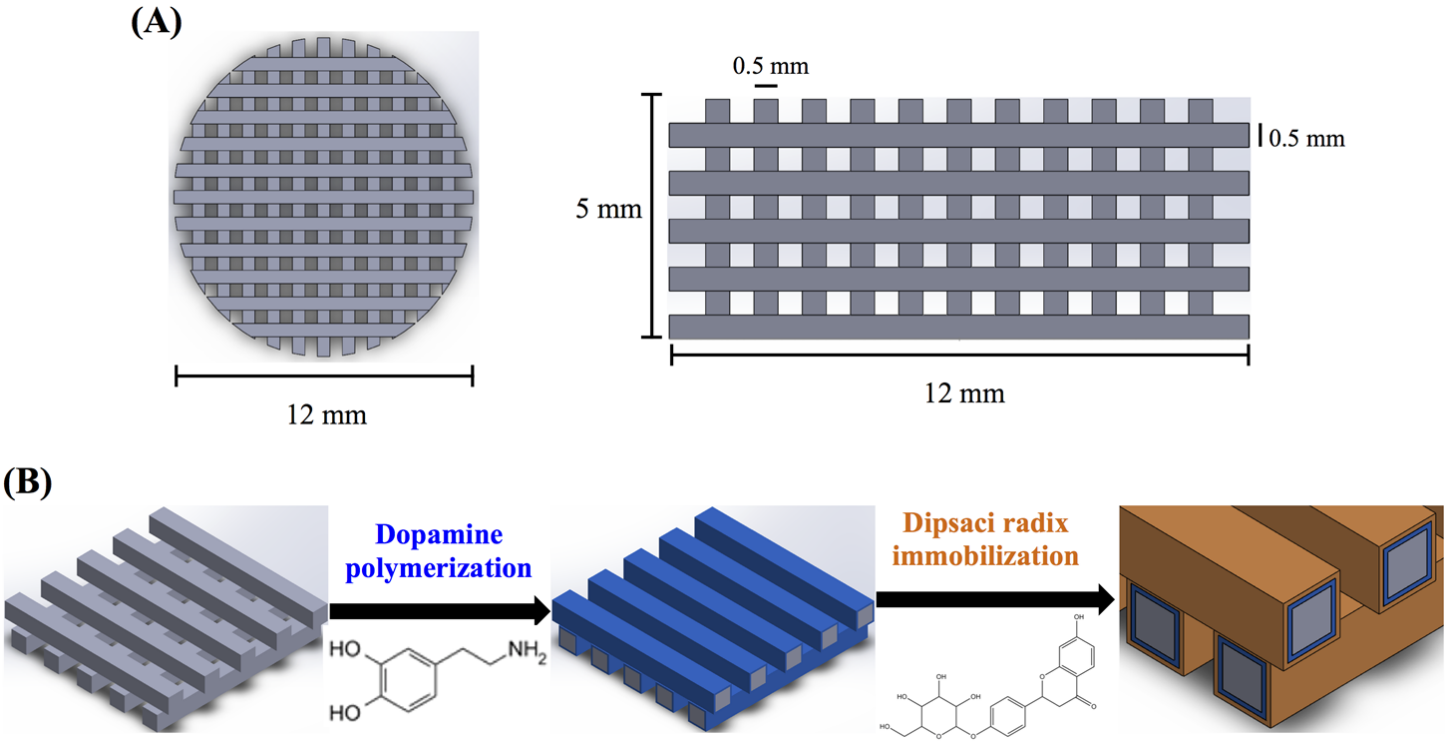
The (**A**) top view and side view of 3D printed poly(lactic acid) (PLA) scaffold; (**B**) Schematic illustration of dopamine-assisted immobilization of XD on surfaces.

The 3D printed scaffolds were designed using AutoCAD 2013 software (Autodesk, Inc., San Rafael, CA, USA). The 3D CAD model was created using software and saved as stereolitography (.stl) file allowing direct import into the printer software. In the printer, a cartridge is installed to supply the feedstock PLA filament (Pitotech, Changhua City, Taiwan) into the cube 3D printer (Pitotech, Changhua City, Taiwan), where the filament is drawn and melted (temperature: 200 °C) and extruded through the nozzle (size: 0.2 mm) to deposit beads of layer which has the ability to melt process up to three separate filaments in diameter 0.5 mm, gap 0.5 mm. The layer thickness can be set to 0.5 mm for fine details and good print quality (Figure 1). The height and diameter of scaffold were 5 mm and 12 mm, respectively. In addition, the resolution of Z-axis was 0.1 mm.

### 2.2. Preparation of Xu Duan (XD) Powder and Immobilization

The XD (*Dipsacus asper Wall.*) was obtained from a local Chinese medicine/herb store in Taiwan, and the identity confirmed as XD by experts in pharmacognosy [27]. Aqueous XD extracts were prepared by standardized procedures. Briefly, a 50 g ground specimen of XD was added to 500 mL of distilled water and boiled under reflux for 2.5 h. Then, the extracts were filtered to remove insoluble debris and concentrated under 50 °C using vacuum evaporation. Finally, the XD powder was freeze-dried in this experiment [27]. Then, the PLA-PDA coated scaffolds were immersion in XD solution (1 and 2 mg/mL) for 24 h under 25 rpm shaker at room temperature, followed by several rinses with deionized water (Figure 1).

### 2.3. PDA Coating

The deposition of PDA onto PLA scaffold was conducted via direct immersion coating. All PLA scaffolds were rinsed with deionized water before PDA immersion. For the PDA coating, the scaffolds were soaked in a dopamine solution (2 mg/mL in 10 mM Tris, pH 8.5) under 25 rpm shaker at room temperature. PLA scaffolds were soaked in PDA solution at room temperature for 12 h, followed by several rinses with deionized water. The elemental compositions of the PDA-coated scaffolds were characterized with an electron spectroscope for chemical analysis (ESCA, PHI 5000 VersaProbe, ULVAC-PHI, Kanagawa, Japan). The concentration of elements (N, O, and C) was given in atomic percent. The sample number of all experiments was six and done in triplicate. We evaluate the morphology of scaffold by scanning electron microscopy (SEM; JEOL JSM-7401F, Tokyo, Japan).

### 2.4. In Vitro Release of Xu Duan

The release of Xu Duan was analyzed after immersing scaffolds in 2 mL of Dulbecco’s Modified Eagle Medium (DMEM, Caisson, North Logan, UT, USA) at 37 °C for different time-points. The count of Xu Duan in medium was measured using the Bio-Rad DC Protein Assay kit. The DMEM without materials was used as the control.

### 2.5. Human Bone-Marrow Mesenchymal Stem Cell Culture

The human bone-marrow mesenchymal stem cells (hBMSCs) were obtained from ScienCell at passage 3, and cells were expanded in culture medium until passages 3–8 (P3–P8). The sample size for all material groups and the tissue culture plastic control (Ctrl) was six. The culture medium consisted of Dulbecco’s modified Eagle’s medium (DMEM, Caisson) with 10% fetal bovine serum (GeneDireX), 1% penicillin/streptomycin (Caisson). The osteogenesis differentiation media was normal medium contained 10^−8^ M dexamethasone, 0.05 g/L L-Ascorbic acid and 2.16 g/L glycerol 2-phosphate. In addition, the angiogenesis induction reagent had 3% FBS, 1% penicillin /streptomycin, and 20 ng/mL vascular endothelial growth factor were dissolved with media.

### 2.6. Cell Adhesion, Proliferation and Cytotoxicity

Cell (10^4^ cells/mL) were directly cultured on scaffolds for various time periods at 37 °C in a 5% CO_2_ atmosphere. After culturing, cell viability was estimated using the PrestoBlue^®^ assay. Briefly, at the end of time point, the scaffolds were change to new well and PBS washed the material. The media with a 1:9 ratio of PrestoBlue^®^ in fresh media was filled with well and incubated for 30 min at 37 °C. Then, the mixture in each well was transferred to new ELISA plate and analyzed by the spectrophotometer with wavelength of 570 nm, and a reference of 600 nm. The hBMSCs cultured on the tissue culture plate without the scaffolds was used as a control (Ctl). In addition, the activity of lactate dehydrogenase (LDH) in the medium released from hBMSCs cultured on the specimens was used as an index of cytotoxicity. Briefly, hBMSCs were cultured on the scaffolds at an initial density of 10^4^ cells per scaffold for 1, 3, and 7 days, then the culture medium was collected and centrifuged, and the LDH activity in the supernatant was determined by spectrophotometer measured at 490 nm absorbance according to the manufacturer's instruction (Sigma-Aldrich, St. Louis, MO, USA).

### 2.7. Cell Morphology

We evaluate the cell adhesion on scaffold by scanning electron microscopy (SEM; JEOL JSM-7401F, Tokyo, Japan). The specimens were washed three times with PBS and fixed in 1% glutaraldehyde (Sigma-Aldrich, St. Louis, MO, USA) for 2 h after cell seeding. The specimens were then dehydrated using a graded ethanol series for 15 min at each concentration and dried at 60 °C in the oven. The dried specimens were mounted on stubs, coated with gold, and viewed using SEM.

### 2.8. Western Blot

The hBMSCs were lysed in NP-40 buffer on ice for 1 h and the suspensions were centrifuged at 14,000 g. The cell lysates were separated by SDS-polyacrylamide gel electrophoresis, and then transferred to nitrocellulose membranes. The membrane blocked in 3% bovine serum albumin (BSA) for 30 min and then immunoblotted with the primary anti-FAK, anti-phosphor-FAK, and β-actin (GeneTex, San Antonio, TX, USA) for 3 h, then washed three times by the tris-base saline buffer containing 0.05% Tween-20. A horseradish peroxidase (HRP)-conjugated secondary antibody was subsequently added and the proteins were visualized with enhancement using enhanced chemiluminescent detection kits (Invitrogen, Carlsbad, CA, USA). The stained band was scanned and quantified using a densitometer (Syngene bioimaging system, Frederick, MD, USA) and ImageJ software (National Institutes of Health, Bethesda, MD, USA). The protein expression level was normalized to the β-actin for each group.

### 2.9. Real-Time PCR

The bone-related gene [collagen I (COL), alkaline phosphatase (ALP), osteopontin (OPN), and osteocalcin (OC)] of hBMSCs was cultured at a density of 10^4^ cells per sample for different time-points (7 and 14 days). Total RNA of hBMSCs cultured on specimens was extracted by TRIzol reagent (Invitrogen). RT-qPCR primers were selected from the NCBI Sequence database were list in Table 1. SYBR Green qPCR Mixes were used for target mRNA and analyzed by ABI Step One Plus system (Applied Biosystems, Foster City, CA, USA).

**Table 1:** Primer pairs used in the qRT-PCR.

Genes	Primer Sequences
COL	Forward: 5’-AGAACAGCGTGGCCT-3’
	Reverse: 5’-TCCGGTGTGACTCGT-3’
ALP	Forward: 5’-TCAGAAGCTAACACCAACG-3’
	Reverse: 5’-TTGTACGTCTTGGAGAGGGC-3’
BSP	Forward: 5’-TCACCTGTGCCATACCAGTTAA-3’
	Reverse: TGAGATGGGTCAGGGTTTAGC-3’
OC	Forward: 5’-GCAAAGGTGCAGCCTTTGTG-3’
	Reverse: GGCTCCCAGCCATTGATACAG-3’
18S	Forward: 5’-CGGAACTGAGGCCATGATTAAG-3’
	Reverse: 5’-GTATCTGATCGTCTTCGAACCTCC-3’

### 2.10. Alizarin Red S Stain

The Ca deposition was considered after two weeks by Alizarin Red S-stained as described in a previous study [28,29,30]. In briefly, hBMSCs were fixed by paraformadedyde (Sigma-Aldrich, St. Louis, MO, USA) in PBS for 20 min and then incubated with 0.5% Alizarin Red S (pH 4.0, Sigma-Aldrich, St. Louis, MO, USA) for 20 min in the shaker (30 rpm). In addition, all specimens were immersed with 1.0 mL of 5% SDS in 0.5N HCl for 0.5 h at room temperature, following which the tubes were centrifuged at 6000 rpm for 15 min and the mixtures were transferred to the 96-well plate to quantify the calcified nodules.

### 2.11. Angiogenesis-Related Protein Analysis

Ang-1 and vWF proteins secreted form cells were analyzed by ELISA kits (Abcam, catalog no. ab99970 and ab108918, Cambridge, MA, USA). Briefly, hBMSCs were seeded on materials for different time points, and proteins from whole cell lysates were collected and analyzed using the ELISA kit according to the manufacturer’s instructions.

### 2.12. Statistical Analysis

Analysis was performed using SPSS software. All the data were expressed as means ± standard deviation (SD) and were analyzed using one-way ANOVA with a Scheffe’s multiple comparison test. A p-value < 0.05 was considered statistically significant.

## 3. Results and Discussion

### 3.1. Characterization of PLA/PDA/XD Scaffolds

**Figure 2:**
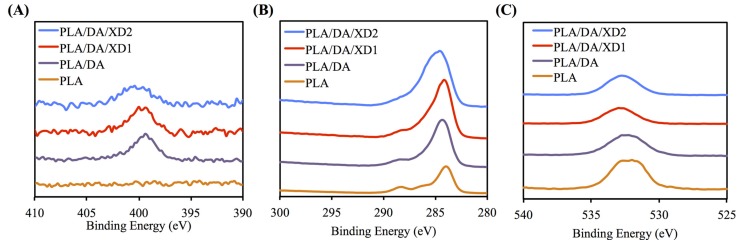
XPS (**A**) N1s; (**B**) C1s; and (**C**) O1s high-resolution spectra obtained on PLA scaffolds after coating with dopamine.

**Figure 3:**
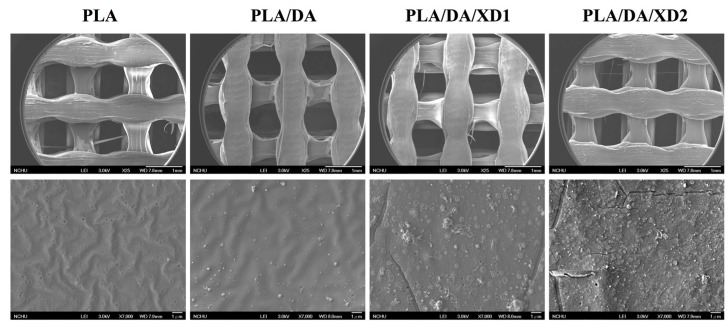
SEM images of PLA scaffold coated with DA and XD.

The PDA coated-layer on PLA scaffolds is based on next two steps: oxidation of DA was oxidized and form 5,6-dihydroxyindole (DHI) that in turn undergoes oligomerization followed by the simultaneous precipitation on PDA layer [31]. Table 2 shows a clear difference between the elemental composition of PLA scaffolds before and after dopamine coating, which show a significant increase in both the carbon and the nitrogen contents and a significant decrease in the oxygen content. As expected, it was observed that elevated amount of DA, resulted in the reduction of O1s, from 33.92% to 25.14%, along with increased concentrations of C1s and N1s, from 66.08% to 71.65% and from 0% to 3.51%, respectively. The oxygen content increased and nitrogen content decreased with increasing XD concentration in the solution. In addition, PDA and XD on PLA are also supported by the XPS O1s high-resolution spectra (Figure 2C). The photoelectron peaks of the PDA coating appear along with emergence of N1s (Figure 2A) and C1s (Figure 2B) at 400 and 285 eV. After PDA coating, the carbon and nitrogen contents were much greater than those seen with the untreated PLA, indicating PDA deposition on the substrate. It is worth noting that the surface oxygen and carbon contents of the PDA-coated PLA were still much higher than the theoretical atomic composition of the PDA, suggesting that the elemental content of the underlying PLA was still dominant and contributed to the overall elemental composition of the surfaces. Moreover, the PDA coating was less than 10 nm thick, the depth limit of ESCA. The PLA scaffolds exhibit smooth surfaces and a uniform shape. However, PDA also appeared to be coated homogeneously all over the surfaces. Our results are consistent with several previous reports, in which PDA was coated on different substrates [32,33,34]. The morphology of the PLA scaffold before and after coating is shown in Figure 3. The PLA scaffolds exhibit smooth surfaces and a uniform shape. However, PDA also appeared to be coated homogeneously all over the surfaces.

**Table 2:** Surface chemical composition of XD immobilized on polydopamine (PDA)-coated PLA scaffolds by XPS.

Code	O1s (%)	C1s (%)	N1s (%)
Dopamine	19.21	70.33	10.46
XD	27.69	72.31	N.A.
PLA	33.92	66.08	N.A.
PLA/DA	25.14	71.65	3.51
PLA/DA/XD1	24.33	72.79	2.88
PLA/DA/XD2	27.05	71.33	1.62

### 3.2. In Vitro Release of XD

**Figure 4:**
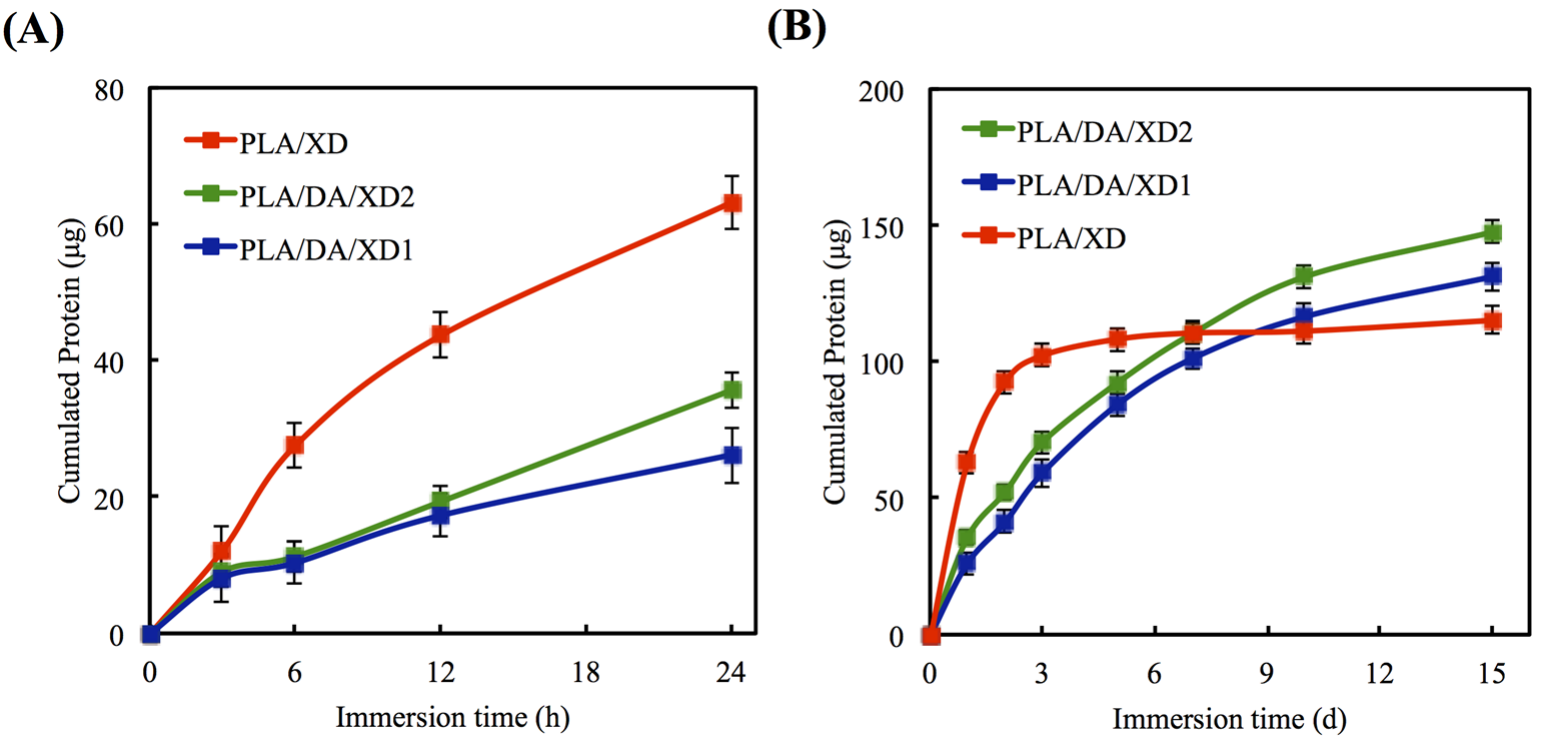
Release profile of XD from PLA scaffolds in DMEM for (**A**) short and (**B**) long times. The values shown are means ± standard errors for all the assays.

The *in vitro* release profiles of XD from PLA/PDA are shown in Figure 3. In uncoated PLA scaffold, XD of the initial burst release is taken, and 63.2 μg and 92.6 μg XD released at 24 and 48 h, respectively (Figure 4A). However, PDA-coated specimens released 41.2 μg and 52.2 μg XD from PLA/DA/XD1 and PLA/DA/XD2 over a period of two days, respectively, and XD sustained released until after 14 days (Figure 4B). Regarding the release rate of XD from the scaffolds, the results show that the rate of XD release from PDA-coated specimens are in accordance with an earlier report [35]. The profiles of composites degradation and XD released were similar. Thus, we hypothesize that the primary and major mechanism of XD release from the pure PLA scaffolds during the first 48 h is by desorption from the composites’ surface [36,37,38]. After soaking for long time, the XD released from PDA-coated PLA scaffolds was more and stable. It is conceivable that the catechol side chain in the structure of PDA that is known as an active group to play a possible role in supplying adsorption sites for hydrogen bonding that cause to the growth factor/PDA coating [39]. The effective concentrations of XD for cell behavior were determined in several previous studies [22,25,40]. XD was verified to have adverse effects on hard tissue regeneration in mice at concentrations higher than 50 mg/kg [41]. Recently, Yao *et al.* showed 10 μg/mL of XD immobilized on bioceramic had the ability to promote new hard tissue formation [27].

### 3.3. Cell Adhesion and Morphology

**Figure 5:**
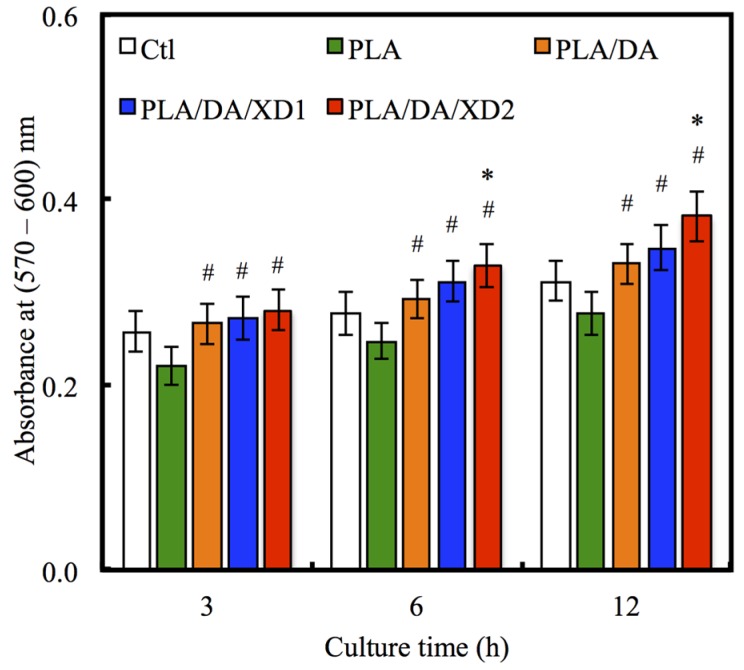
The adhesion of hBMSCs cultured with various specimens for different time points. The values shown are means ± standard errors for all the assays. “#” indicates a significant difference (*p* < 0.05) compared to PLA; “*” indicates a significant difference (*p* < 0.05) compared to PLA/DA.

**Figure 6:**
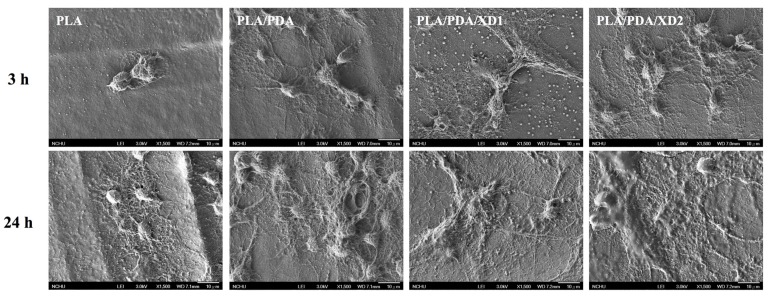
The morphology of hBMSCs adhered on PLA/PDA/XD scaffolds for 3 and 24 h.

**Figure 7:**
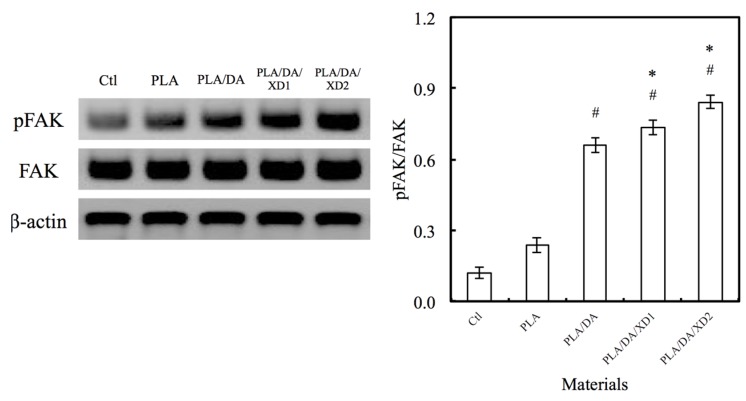
The pFAK expression of hBMSCs cultured on various specimens for 3 h. The values shown are means ± standard errors for all the assays. “#” indicates a significant difference (*p* < 0.05) compared to PLA; “*” indicates a significant difference (*p* < 0.05) compared to PLA/DA.

The facilitation of cell adhesion on the PDA-coated PLA scaffolds was confirmed and observed by PrestoBlue^®^ (Figure 5) and SEM (Figure 6). Figure 5 exhibits the significantly higher number (*p* < 0.05) of hBMSCs is cultured for the PDA/XD hybrid materials than the PLA for all time, consistent with the results of SEM. For example, PLA/DA/XD2 showed a significant enhancement of 27%, 33%, and 39% than uncoated PLA scaffolds after short time-points for cell cultured, respectively. Moreover, PLA/DA/XD2 displayed significantly greater (*p* < 0.05) cell adhered than PLA/DA at 6 and 12 h culture. PLA/DA/XD2 displayed the increase of approximately 21% in the cell proliferation compared with the PLA/DA on the first 12 h cultured. When the hBMSCs were seeded onto PLA substrates for 3 h, the cells barely adhered and spread, whereas the cells cultured on PDA/PLA exhibited normal adhesion. At 3 h, cells adhered to the PLA/DA/XD1 and PLA/DA/XD2 surfaces were spread out, with round cells attaching to the PLA and PLA/XD surfaces. In a previous study, we proved that PLA materials affected the morphology and mineralization of bone cells [42]. In the cases of extracellular matrix components (e.g., collagen) and polycations (e.g., poly-lysine), the improvement in cell adhesion is dependent on the type of materials and cell lines [31], but our strategy using PDA ad-layer could increase the cell adhesion efficiency on different types of substrates and cell lines. In addition, the effect of substrates on pFAK expression by cells was also examined, and there was an increase in pFAK production with increasing XD content (Figure 7). pFAK expression was significantly higher (*p* < 0.05) on the substrates with XD2 immobilization than on the pure PLA after cells seeding for 3 h. In cell adhered stage, several extracellular matrix protein components were secrete by hBMSCs, such as cellular FN or collagen on the materials that improve cellular behavior [42]. Col I contain numerous cells binding sites that are known to adsorb on PDA, and thus mediate cell adhesion. The recognition and binding of the integrin receptors to substrates initiate focal adhesions and subsequently activate FAK by autophosphorylation, the degree of which may consequently influence cell spreading, proliferation, and differentiation [42].

### 3.4. Cell Proliferation

**Figure 8:**
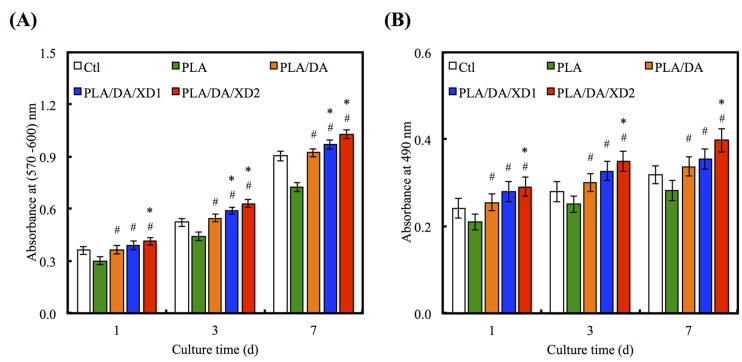
(**A**) PrestoBlue^®^ assay and (**B**) LDH assay of hBMSCs cultured on various specimens for different time points. The values shown are means ± standard errors for all the assays. “#” indicates a significant difference (*p* < 0.05) compared to PLA; “*” indicates a significant difference (*p* < 0.05) compared to PLA/DA.

The increase in cell adhesion may be directly related to the improvement of surface hydrophilicity [43] and functional groups (e.g. OH^−^, NH_2_^−^) [18]. To consider the effects of XD and PDA on cell proliferation of hBMSCs, various groups were evaluated at different time-points (Figure 8A). The result shows the proliferation of hBMSCs on all PDA-coated PLA scaffold was significantly higher than PLA (*p* < 0.05) at all culture time-points. The proliferation rate of hBMSCs gradually increased along with the amount of XD on PDA-coated PLA that indicated a significant difference compared with PDA-coated PLA specimens (*p* < 0.05). For example, XD2 showed an increase of approximately 16.1% and 19.2% compared to PLA/DA on day 3 and 7. The number of hBMSCs on XD1 and XD2 was even higher than that seen on Ctl, the standard cell culture vessel material. To evaluate the cytotoxic effect of the different specimens, the amount of LDH released to hBMSCs cultured on the presence of the different scaffolds was determined after 1, 3 and 7 days (Figure 8B). The results for the DA and XD coated scaffolds indicated that no cytotoxic agent was released. It suggesting higher cell viability and better biocompatibility for DA and XD coated scaffolds.

### 3.5. Osteogenesis Differentiation

**Figure 9:**
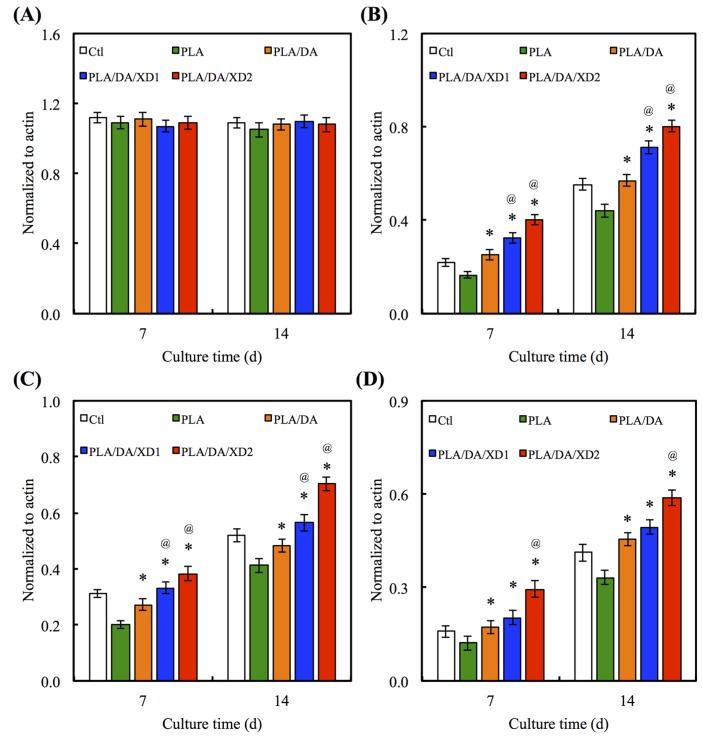
(**A**) Col; (**B**) ALP; (**C**) BSP and (**D**) OC gene expression in the hBMSCs were cultured on the various specimens for 7 and 14 days. The values shown are means ± standard errors for all the assays. “@” indicates a significant difference (*p* < 0.05) compared to PLA; “*” indicates a significant difference (*p* < 0.05) compared to PLA/DA.

There is no obvious difference for the Col gene expression between all specimens at day 7 and 14 (Figure 9A). However, the bone-related gene expression for ALP, BSP and OC of hBMSCs on PLA/PDA/XD1 and PLA/PDA/XD2 are obviously higher than on specimens without XD (Figure 9B–D). Interesting, the expression of ALP, BSP and OC of cells on PLA/PDA/XD2 elicited a significant (*p* < 0.05) increase of 17%, 25%, and 20% compared with PLA/PDA/XD1 on day 14. ALP is an early marker of osteogenesis differentiation, and it is generally accepted that an increase in the specific activity of ALP in bone cells reflects a shift to a more differentiated state [21]. BSP and OC are later makers of osteogenic differentiation.

### 3.6. Calcium Deposition

**Figure 10:**
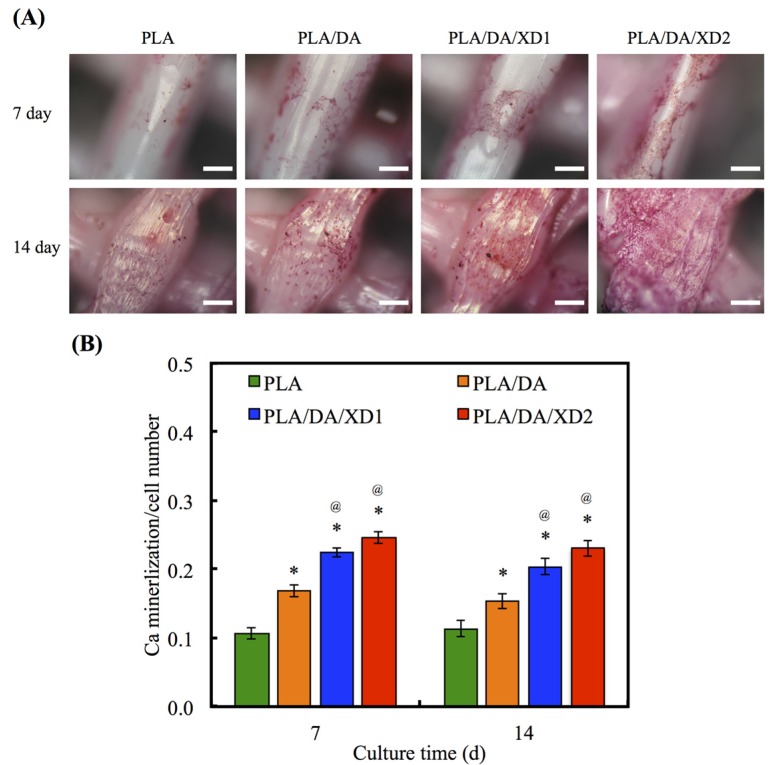
(**A**) Alizarin Red S staining and (**B**) quantification of calcium mineral deposits of hBMSCs cultured on various scaffolds for 7 and 14 days. The values shown are means ± standard errors for all the assays. “*” indicates a significant difference (*p* < 0.05) compared to PLA; “@” indicates a significant difference (*p* < 0.05) compared to PLA/DA.

The calcium deposition of hBMSCs to form a mineralized matrix is important for the establishment of biomaterials suitable for hard tissue regeneration. The aim of the mineralization assay is to evaluate and show the effects of XD released from PDA-coated PLA scaffolds on Ca matrix deposition following analysis by Alizarin Red S-stained to recognize Ca deposition, shown in Figure 10. The photographs of Alizarin red S-stained hBMSCs were seeded on various scaffolds for period time-points. After cultured for 7 and 14 day, the results showed a little stained was observed on PLA surface, whereas more Ca deposition was formed on PDA-contained surfaces. Quantification of the Alizarin Red S assay displayed that the Ca content increased with more cultured time-points. It has been proved that PDA-coated is useful for promoting hBMSCs’ behavior, containing osteogenesis, adhered and biocompatibility of hydrophobic materials. In addition, the present results proved the beneficial influences of XD immobilization in the PDA layer on osteogenic differentiation, and XD promotes cells in higher ALP activity and a trend toward higher mineral deposition [27].

### 3.7. Angiogenesis

**Figure 11:**
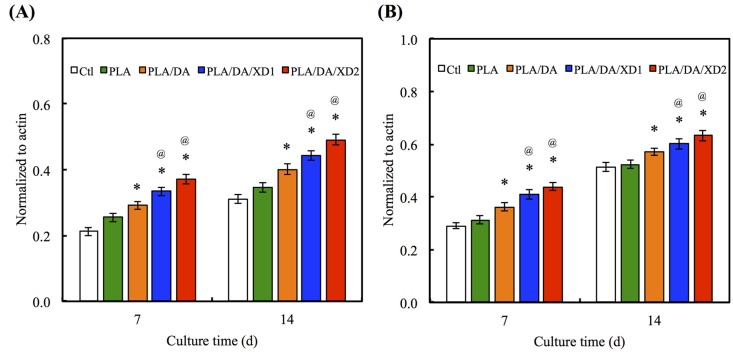
The protein expression of (**A**) Ang-1 and (**B**) vWF of hBMSCs cultured on scaffolds for different days. The values shown are means ± standard errors for all the assays. “*” indicates a significant difference (*p* < 0.05) compared to PLA; “@” indicates a significant difference (*p* < 0.05) compared to PLA/DA.

Angiogenesis is necessary for successful bone formation and regeneration. The Ang-1 and vWF secreted from hBMSCs seeded on scaffolds were analyzed (Figure 11). ELISA analysis showed that hBMSCs on pure PLA scaffold group expressed the Ang-1 and vWF protein at basal levels, similarly to the Ctl group. In contrast, in the PDA-coated groups, expressions of the angiogenic protein were significantly increased (*p* < 0.05) compared with Ctl and PLA. Ang-1 is an important protein in blood vessel formation at later stages, such as the stabilization of the endothelial sprout and its interaction with pericytes. Moreover, it could also decrease VEGF-mediated vascular permeability [44,45,46]. vWF is the large glycoprotein of the vascular endothelial matrix that binds its receptors on platelets when the molecule is perturbed by binding to ECM [47,48]. Several studies have proved that stem cells can release several pro-angiogenic molecules like VEGF to recruit inflammatory and progenitor cells to make the wound tissue microenvironment conducive for vessel formation and subsequent healing [49]. Furthermore, PDA specifically regulates the vascular endothelial growth factor-induced phosphorylation of vascular endothelial growth factor receptor-2 during the earliest steps of the angiogenic process [50]. Therefore, our results suggest that the production of angiogenesis factors by XD/PDA-coated-PLA-stimulated cells is more advantageous than the local delivery of a single angiogenic protein.

## 4. Conclusions

In summary, we successfully immobilized XD on fabricated bio-inspired PDA-coated PLA scaffolds, which improve cell proliferation and prolong the XD released. Furthermore, the XD immobilization on PDA-coated PLA scaffolds allow hBMSCs to grow and differentiate better than on the PDA-coated PLA scaffolds. The XD immobilization on scaffolds was also demonstrated to induce osteogenesis and angiogenesis differentiation. Therefore, our results prove that this simple, bio-inspired surface modification and XD immobilization of the organic PLA scaffolds is a very promising tool to regulate stem cell behavior, and may serve as an effective stem cell delivery carrier for bone tissue engineering.
